# Factors affecting the use of medicinal plants by migrants from rural areas of Brazilian Northeast after moving to a metropolitan region in Southeast of Brazil

**DOI:** 10.1186/s13002-018-0270-3

**Published:** 2018-11-22

**Authors:** Perla Carvalho Romanus, Fúlvio Rieli Mendes, Elisaldo de Araújo Carlini

**Affiliations:** 1 Department of Psychobiology, UNIFESP, Rua Botucatu, 862, 1° andar, prédio Ciências Biomédicas, Vila Clementino, São Paulo, SP 04023-062 Brazil; 2 Center for Natural and Human Sciences, UFABC, Rua Arcturus, 03, Sala 236, Bloco Delta. Bairro Jardim Antares, São Bernardo do Campo, SP 09606-070 Brazil; 3 Department of Preventive Medicine, UNIFESP, Rua Botucatu, 740, 4° andar. Bairro Vila Clementino, São Paulo, SP 04023-900 Brazil

**Keywords:** Medicinal plants, Migration, Adaptation, Urban ethnomedicine, Bororé Peninsula, Cross-cultural adaptation

## Abstract

**Background:**

Ethnopharmacological studies about migrants reveal a dynamic process of knowledge and use of medicinal plants. In this study, we sought to elucidate quantitative and qualitatively the main factors influencing the use of medicinal plants by migrants from rural areas to an urban region in Brazil with traces of remnant natural vegetation.

**Methods:**

Seven Northeastern individuals who migrated to the Southeastern Region of Brazil (Bororé Peninsula, in the city of São Paulo) were selected to participate in semi-structured interviews regarding the use of medicinal plants throughout their lives, and indicated an inhabitant in their hometown that would be able to accompany the field collections in each area. Socioeconomic, educational, family structure, and use of Western medicine data were provided during interviews with the individuals from their hometowns. Plant samples cited by the interviewees were collected both at the current place of residence and in their hometowns.

**Results:**

The participants cited 131 plants and 315 recipes, being the main indications related to the gastrointestinal system, respiratory problems, and pain and inflammatory processes. We observed that most plant uses were maintained after migration. Higher percentages of maintenances and incorporations in plant uses occurred to exotic species, while replacements happen mainly to native plants. The introduction of new species into the migrants’ therapeutics occurred mainly by observations of organoleptic similarities between the substituted plant and the incorporated species, conversations with neighbors, and contact with the television and print media. In addition, the public health system allowed the interviewees access to prophylactic drugs, leading to the discontinuation of certain recipes used in endemic diseases.

**Conclusion:**

Migrants were exposed to information about new plants and their uses, new diseases, and socioeconomic and cultural differences that impacted their use of medicinal plants. Although migration to a more developed city facilitated access to public health and education, on the other hand, it made access to fresh medicinal plants difficult, causing some medicinal plants to be replaced or ceased to be used.

**Electronic supplementary material:**

The online version of this article (10.1186/s13002-018-0270-3) contains supplementary material, which is available to authorized users.

## Background

Ethnopharmacological research regarding migrants reveals a dynamic process of knowledge and use of medicinal plants. Despite adapting or incorporating new treatments for their illnesses, migrant subjects also preserve part of their past culture. Some of the factors that can influence this process are the reason for migration, social status, education, personal habits, lifestyle, and differences in the degree of development between the regions of origin and destination [1–4].

Numerous studies have evaluated the impact of the cultural syncretism on the adaptation of different groups of migrants, for example, people that migrated from Albania or Senegal to Italy [5, 6], from Haiti, Europe, and Africa to Cuba [7, 8], from South Asian or South American countries to UK [3, 9–11], from Macedonia to Albania [12], from Poland to Argentina [13, 14], and from Austria to Australia, Brazil, or Peru [15]. This adaptation is also observed in studies with nomad people [16] and migrants from geographically and culturally distinct regions of the same country, such as Brazilian subjects who migrated from the Northeastern Region to the Amazon region of the Acre and Purus rivers [17] and from the northeastern to southeast metropolitan region of São Paulo [18]. The migrants usually cherish and uphold values and traditions of their hometowns, such as festive dates, cuisine, music, local dialects, and the medicinal plants they already knew. At their new destination, they are introduced to novel uses for these plants, in addition to introducing their knowledge to local therapeutics as well [1, 19]. Additionally, they are exposed to a new environment where the acquisition of new species and the abandonment of certain plants of their therapeutic resource take place [1, 2].

Migration from rural to urban areas in metropolitan centers [18] or immigration from developing countries to big cities in developed countries have been previously studied [3, 4, 6, 9, 10, 20]. When migrants move to regions that have preserved vegetation or remain in contact with people from their hometown, the likelihood of maintaining the use of medicinal plants is higher, despite cultural and climatic adaptations [1, 21].

In this study, we sought to investigate, using quantitative and qualitative data, how the migration from rural areas of the Brazilian Northeast to the Bororé Peninsula (a community belonging to São Paulo metropolitan region, but with remaining forest areas) influenced the dynamics of the use of medicinal plants among the migrant subjects. In order to better understand the rationale behind the decision of whether or not to use medicinal plants after migration, ethnopharmacological and ethnographic data were collected both at the São Paulo site and on informants’ cities of origin. Our hypothesis was that the migrants would maintain the use of the species that are also available in the host environment (new city) and discontinue the use or replace those not available in the metropolitan region.

## Methods

### Locations (study areas)

The Bororé Peninsula is located in the Billings Hydrographic Basin, which occupies a territory of 582.8 km^2^, located in the southeast portion of the metropolitan region of São Paulo, Southeast Brazil. Contrasting with the other regions of the city, it has low human occupation, with few buildings and access by ferry boat. It contains a large area of secondary Atlantic Forest in middle and advanced stages of regeneration [22].

During the survey period, the community of the Bororé Peninsula had approximately 2500 inhabitants (data obtained through the local Family Health Unit). Many inhabitants came from migratory flows in the 1960s and 1970s. There was one Basic Health Unit and one public school in the Bororé Peninsula, as well as electricity and telephone network, but there was no water supply, resulting in the need for residents to build artesian wells. Residents of the Bororé Peninsula could schedule weekly medical appointments at the Basic Health Unit, while elderly patients, pregnant women, and sick people with most serious health state received authorization to conduct tests and medications at home through government health employee.

**Fig. 1:**
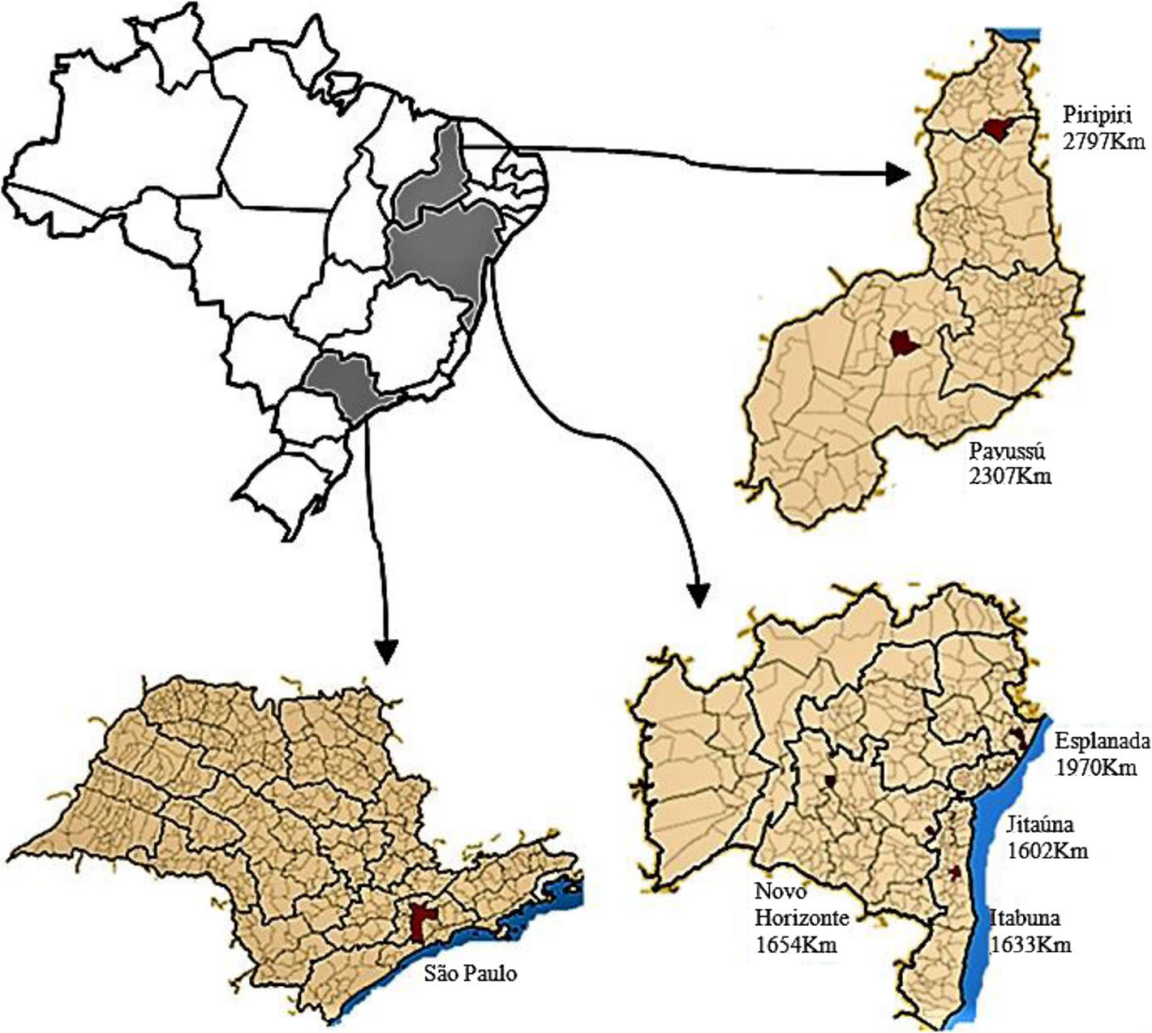
Location of the cities of origin of the interviewees, indicating their distances to São Paulo city

**Fig. 2:**
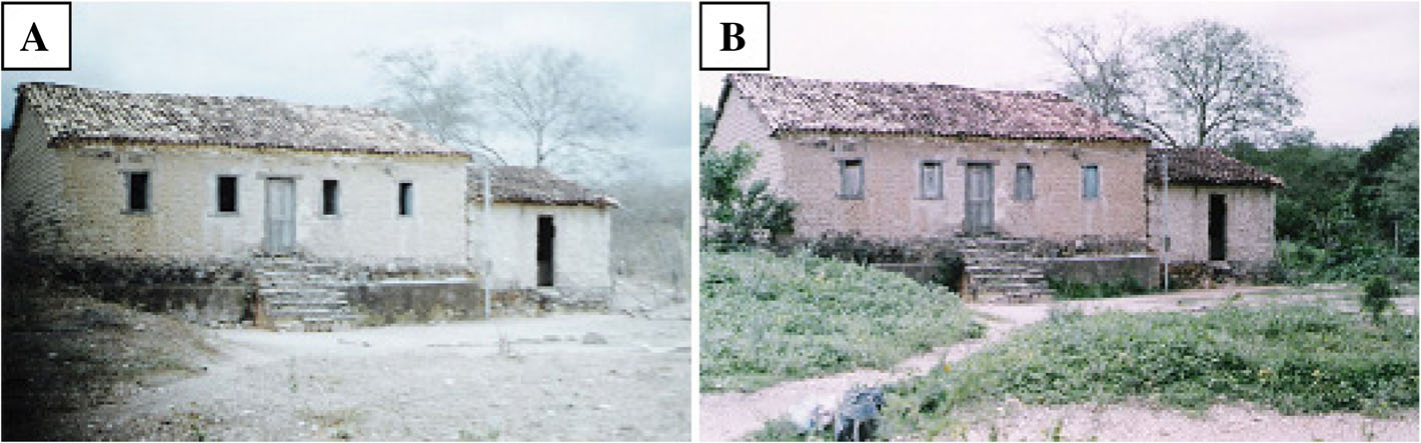
Residence in a rural area of the city of Novo Horizonte, state of Bahia. **a** Summer equals dry season. **b** Winter equals rainy season

The informants’ hometowns are located in Bahia and Piaui (Northeast states of Brazil). Esplanada, Jitaúna, and Itabuna (in the state of Bahia) present the Atlantic Forest as a natural biome, while Novo Horizonte (Bahia), Piripiri, and Pavussú (in the state of Piauí) are located in regions where the prevalent biome is the Caatinga (dryland) (Fig. 1). The Caatinga is represented by a rainfall regime that, in the regional culture, encompasses two distinct seasons: summer (dry season) and winter (rainy season) (Fig. 2).

Basic sanitation, public health facilities, basic education (primary and secondary schools), public transport, and access to electricity in rural areas were precarious or absent in all visited cities. Although Itabuna city was an exception for most of the aforementioned issues, basic sanitation was absent in the visited rural area at the margin of the forest. Another concern was the presence of mining in new Horizonte, where miners could be seen working without any supervision or security. Extreme poverty was observed in some places, but according to the interviewees, it had been recently diminished as a consequence of the federal basic income transfer policy called “Bolsa Família” which pays a small salary for poor families.

### Selection of participants, interviews, and sample collection

Approximately 300 houses were visited in the Bororé Peninsula between July 2005 and August 2006. During this time, we collected general information about the local community and set some key-informants (community leaders) to assist in selecting the study participants. For this study, we selected people who migrated from the Northeast Region of Brazil and met the following criteria: (a) were considered specialists in medicinal plants by their neighbors in the Bororé community; (b) used native plants regularly in their hometown; and (c) were able to indicate at least one close person (friend or relative) who could assist in ethnographic research and plant collection in their hometown. Seven people with these characteristics were selected (data summarized in Table 1).

**Table 1:** Personal data of the interviewees

Interviewee^1^	Age (year)	Gender	Origin	Hometown biome	Trigger for migration	Time after migration (years)	Scholar degree	Profession	Grows medicinal plants at the yard?
BA1	68	Male	Itabuna, BA	Atlantic Forest	Family	15	Informal literacy	Housekeeper	Yes
BA2	53	Female	Novo Horizonte, BA	Caatinga	Curiosity	15	Illiterate	Housewife	Yes
BA3	62	Male	Jitaúna, BA	Atlantic Forest	Financial improvement	20	Elementary school	Housekeeper	No
BA4	49	Female	Jitaúna, BA	Atlantic Forest	Financial improvement	20	Elementary school	Unemployed	No
BA5	55	Female	Esplanada, BA	Atlantic Forest	Curiosity	15	Elementary school	Housekeeper	Yes
PI1	48	Female	Piripiri, PI	Caatinga	Financial improvement	20	Elementary school	House maid	Yes
PI2	63	Male	Pavussu, PI	Caatinga	Financial improvement	20	Informal literacy	Gardener	Yes

^1^*BA* migrants from the state of Bahia, *PI* migrants from the state of Piauí

Survey methods were based on anthropological and botanical concepts [23] to obtain qualitative and quantitative data about the use of medicinal plants. Approximately 40 informal and semi-structured visits and interviews were carried out with the migrants (informants) to obtain their personal data such as age, marital status, main occupation, educational level, religion, family structure, city of birth, migratory journey, motive that led to migration, and housing time. Using field notebooks, guidebooks, and by participant observation method (direct observation) [23, 24], we also obtained data about usual diet, use of the conventional health system, and allopathic medicines, as well as detailed information on each medicinal plant known to the interviewee. We then collected the botanical material, whenever possible, and we registered the plant popular names, physical characteristics, indications (popular uses), used parts, methods of preparation, routes of administration, doses, frequency, contraindications, adverse effects, and any other relevant characteristics. Each indication (containing the part used and method of preparation) was considered one recipe. Some recipes contained two or more species (formulas). The same taxon and recipe would be cited by more than one informant. Plants cited by the informants, but not available in the Bororé Peninsula, were not collected in this phase of the study, but all relevant information was recorded to allow the subsequent localization of the species at the migrant’s cities of origin.

In the second phase of the study (between September and November 2006), the fieldwork was performed at the interviewees’ hometowns, where nine friends or relatives assisted as local guides for botanical collections. Local guides were informed of the popular name, medicinal use, and main morphological and organoleptic characteristics of the plants cited during the interviews in the peninsula of Bororé in order to increase the chances of finding the correct species. Books on Brazilian medicinal flora (see Additional file 1) were consulted in order to obtain data on their geographical distribution and their popular names to correlate with the names cited by the interviewees. This information was useful to facilitate finding the plants in the city of origin of each participant. The plants found and collected during the second phase of the study were subsequently showed to the migrants to confirm that they matched with the cited species.

The material collected in Bororé Peninsula and in the informants’ hometown was identified at the Botany Institute of the State of São Paulo, and the vouchers specimens were deposited at the herbarium of the Federal University of ABC. The website “Flora do Brazil” [25] and several books were consulted to determine if the species were native, naturalized, or exotic (Additional file 1). Plants purchased in supermarkets by the participants were not identified by collection; instead, the botanical species or possible genus was suggested according to the organoleptic properties cited by the respondents, or by the information contained in their commercial packages (when available), as in the case of tea bags.

Considering the context of human migration, the adaptation in the use of medicinal plants was categorized as follows [18, 26]: (1) *maintenance*—when a plant known in the city of origin has its indication kept in the current location of residence; (2) *replacement*—there was an exchange of a medicinal plant used in the hometown for another species used for the same purpose; (3) *incorporation*—new indication for an already known plant or use of a new species for a specific purpose; (4) *discontinuation*—the species ceased to be used because the disease is uncommon in the current place of residence or the migrant preferred the use of allopathic medicine or an industrialized product in place of the plant.

### Quantitative analysis

The data collected was entered in a worksheet containing the plant vernacular name, popular uses, parts employed, method of preparation, route of administration, and whether the plant use was maintained after migration, replaced, discontinued, or if a new plant or indication (recipe) was incorporated at the host place. Each recipe was included as a single line in the table; when two or more participants cited an identical recipe, it was grouped as a single entry, but the total number of citations was counted in the quantitative analysis. After botanical identification, the scientific name, family, and the origin of the species (native/naturalized or exotic) were included in the worksheet. We then classified the popular uses (complaints, ethnomedical indications, and other applications) cited by the informants into 15 categories (adapted from the International Classification of Diseases—ICD-11) [27]. The data generated was used to calculate the informant’s consensus factor (ICF) and index of relative importance (RI), as detailed below.

The level of homogeneity among information provided by the seven participants was calculated using the formula ICF = *N*_ur_ − *N*_t_/(*N*_ur_ − 1), where *N*_ur_ is the number of use reports from informants for a particular plant use category and *N*_t_ is the number of species that are used for that category for all participants [28, 29].

The RI of the main species cited by the informants was calculated by the formula RI = *N*i/*N*c where *N*i is the number of informants that cited this species and *N*c is the total number of citations of the species.

All taxa and recipes were used in the calculation of ICF and RI, independent of being maintained, replaced, incorporated, or discontinued.

### Qualitative analysis

To better understand the main factors that contributed to the dynamics of medicinal plant use among the migrant participants of this study, the first author (Romanus, PC) recorded notes during the interviews in her field notebook and did general observations for further analysis. We subsequently used these notes to qualitatively discuss some strategies adopted by the seven interviewees concerning the use of medicinal plants after their migration. We sought to understand and discuss the most relevant aspects that influenced the dynamics of medicinal plant use, according to the participants’ reports and interviewer perception.

## Results and discussion

### Migrants selection

Only seven people that fulfilled the selection criteria consented to participate in the study, being 4 women and 3 men with average age of 56.9 years old (Table 1). These informants migrated from Bahia and Piauí states (Northeast of Brazil) around 15–20 years before the study, and the biome in their place of origin was Atlantic Forest (interviewees BA1, BA3, BA4, and BA5) and Caatinga (BA2, PI1, and PI2). All the informants stated that they used medicinal plants in their hometowns and still use in the host place, but two (BA3 and BA4) were not able to grow medicinal plants in their yards at the Bororé Peninsula. The informants had only informal literacy or elementary school and, at the time of the interviews, worked as a housekeeper, housemaid, or gardener in their neighborhood. They also informed the main reasons for migration: financial improvement (4 participants), “curiosity about the life in a big city” (2 participants), or to live close to the family (1 participant).

Similarly to what was reported by Garcia et al. [18], the migrants stated that they learned about medicinal plants with relatives in their hometown and acquired new knowledge from books, media, and neighbors after their migration.

### Sample collection and data analysis

The 131 plants cited during the field work at Bororé Peninsula and in the cities of Bahia and Piauí states are shown in Tables 2, 3, 4, and 5. Most taxa were identified, belonging to 51 families, where the most common were Lamiaceae (14 species), Asteraceae and Fabaceae (13 species), and Euphorbiaceae (11 species). The informants cited 315 recipes as part of several indications (popular uses). The plant parts most employed in the recipes were leaves and aerial parts, followed by barks, branches, and seeds, while the most common methods of preparation were as infusion, syrup, maceration, and decoction, respectively. This data corroborates previous reports in the literature, with these botanical parts and methods of preparation being the most cited. According to Gazzaneo et al. [29], communities living near humid forests tend to use plant leaves, while barks and roots are preferred by people living in dry regions because most plants shed their leaves in dry season. In our study, we observed a substantial use of bark and root of Caatinga species.

**Table 2:** Recipes and medicinal plants which uses were maintained by the informants after their migration to Bororé Peninsula

Scientific name (family)/voucher	Popular names	Origin^*^	Popular use/[interviewee]	Plant part (preparation method)	Route
*Aegiphila* sp. (Verbenaceae) PCR 116	Fumo-brabo	UNK	Cough [BA1]	Aerial (syrup)	Oral
*Ageratum conyzoides* L. (Asteraceae) PCR 27, 179	Mentrasto, mentrasto-branco	N	Healing process [BA3, BA4]	Leaf (roast and grind)	Topic
*Allium* cf. *sativum* L. (Liliaceae) [not collected]	Alho (garlic)	E	Catarrh (yellow color) [BA1]	Bulb (grind)	Inhalation
			Vermifuge [PI2]	Bulb (see recipe 1)	Oral
*Aloysia gratissima* (Gillies & Hook.) Tronc. (Verbenaceae) PCR 17	Alfazema, fazema	N	Body ache [BA2]	Branches (infusion)	Topic (bathing)
			To vent the house (pleasant scent of lavender) [BA1]	Branches (infusion)	Topic (bathing)
			To remove black magic [BA1]	Branches (infusion)	Topic (bathing)
			To remove black magic [BA2]	Leaf (see recipe 2)	Inhalation
*Aloysia triphylla* Royle (Verbenaceae) PCR 40	Erva-cidreira, cidreira	E	Sedative, to sleep [BA5, PI2]	Leaf (infusion)	Oral
*Alpinia zerumbet* (Pers.) B.L. Burtt & R.M. Sm. (Zingiberaceae) PCR 237	Leopoldina, água-de-colonha	E	To the heart [BA1]	Leaf (infusion)	Oral
*Amburana cearensis* (Allemão) A.C. Sm. (Fabaceae) PCR 136, 138	Emburana	N	Guts pain due to food [BA1]	Seed (see recipe 3)	Oral
*Anadenanthera macrocarpa* (Benth.) Brenan (Fabaceae) PCR 79, 189	Angico, pau-barbado	N	When you have a bad feeling [BA2]	Bark (bitter)	Oral
			Wound [BA2]	Bark (decoction)	Topic
*Ananas* cf. *comosus* (L.) Merr. (Bromeliaceae) [not collected]	Abacaxi (pineapple)	N	Flu [BA1]	Fruit bark (see recipe 4)	Oral
*Argemone mexicana* L. (Papaveraceae) PCR 182, 254	Carro-santo	E	Inflammation with catarrh [BA4]	Seed (maceration)	Topic
			Bronchitis [BA4]	Seed (maceration)	Topic
Asteraceae (undefined species) PCR 193	Losna	UNK	Abortive and post abortion care [BA2]	Aerial (infusion)	Oral
			Indigestion [BA5]	Aerial (maceration)	Oral
*Bacharis trimera* (Less.) DC. (Asteraceae) PCR 50, 181	Carqueja, carqueja-de-praia	N	Fever [BA1]	Leaf (infusion)	Oral
			For the whole gut [BA1]	Aerial (infusion)	Oral
			Gut [BA3, BA4]	Leaf (bitter)	Oral
			Liver [BA3, BA4]	Leaf (bitter)	Oral
			Kidney [BA3, BA4]	Leaf (infusion or maceration)	Oral
*Bidens pilosa* L. (Asteraceae) PCR 2, 71, 153, 180	Picão	N	Against hepatitis [BA2]	Aerial (infusion)	Topic (bathing)
*Caesalpinia pulcherrima* (L.) Sw. (Fabaceae) PCR 26, 102	Maravilha	N	Eye pain [BA1]	Flower (maceration)	Topic
*Cajanus cajan* (L.) Millsp. (Fabaceae) PCR 12, 22, 32, 103, 161, 162	Andú	E	Body ache [BA2]	Leaf (infusion)	Oral
			Inflamed tooth, toothache [BA5]	Leaf (infusion)	Mouthwash
			Inflammation from inside the body [BA1]	Leaf (infusion)	Oral
			Fever [BA2]	Leaf (infusion)	Oral
			Infection with vaginal discharge [BA2]	Leaf (infusion)	Topic
			Cold [BA2]	Leaf (infusion)	Oral
			“Suvaqueira” (armpit odor) [PI2]	Leaf (maceration)	Topic
			For the baby to be born faster at the time of childbirth [BA1]	Leaf (infusion)	Topic (bathing) and oral
*Cedrela fissilis* Vell. (Meliaceae) PCR 249	Cedro	N	Wound [BA1]	Bark (decoction)	Topic
			Catarrh (yellow color) [BA1]	Bark (decoction)	Inhalation
			Baby teeth popping [BA1]	Bark (decoction)	Mouthwash
*Chenopodium ambrosioides* L. (Chenopodiaceae) PCR 49, 61, 218, 253, 255, 328	Erva-de-santa-maria, mastruz, mentruz	N	“Sentindo por dentro” (feeling sick) [BA1]	Aerial (maceration and add to milk)	Oral
			Worm [BA5]	Aerial (infusion)	Oral
			Contusion [BA1]	Aerial (maceration)	Topic
			Tendon inflammation [PI1]	Aerial (maceration)	Topic
			Pneumonia [PI1]	Aerial (maceration)	Oral
*Citrus* sp. (Rutaceae) [not collected]	Laranja (orange)	E	Fever [PI1]	Leaf (infusion)	Oral
			“Bicheira” (pests, as *tunga penetrans*) [PI1]	Leaf (see recipe 5)	Topic
*Citrus* sp. (Rutaceae) [not collected]	Limão (lime)	E	“Bicheira” (pests, as *tunga penetrans*) [PI1]	Leaf (see recipe 5)	Topic
*Coffea arabica* L. (Rubiaceae) PCR 110	Café	E	Gut motility [BA1]	Seed (see recipe 3)	Oral
*Commiphora leptophloeos* (Mart.) J.B. Gillett (Burseraceae) PCR 220	Emburana, emburana-de-cambão, emburana-macho	N	Gut motility [BA1]	Seed (infusion of toasted seed)	Oral
*Costus spiralis* (Jacq.) Roscoe (Zingiberaceae) PCR9, 53, 221	Cana-de-macaco	N	Difficulty urinating [BA1]	All parts (infusion)	Oral
*Cymbopogon citratus* (DC.) Stapf. (Poaceae) PCR 39, 226, 227, 228, 257	Capim-santo	E	Sedative, to calm down, to sleep [BA1, BA2, BA5, PI2]	Leaf (infusion)	Oral
			Sore throat [BA2]	Leaf (infusion)	Oral
			Fever [BA2]	Leaf (infusion)	Oral
			Flu [BA1]	Leaf (see recipe 4)	Oral
			To remove black magic [BA2]	Leaf (incense)	Inhalation
*Eucalyptus* sp. (Myrtaceae) PCR 203	Eucalipto, eucalipto-verdadeiro	E	Domestic hygiene [PI2]	Leaf (incense)	Inhalation
			Breast congestion [PI1]	Leaf (infusion)	Inhalation
*Eugenia uniflora* L. (Myrtaceae) PCR 15, 57	Pitanga	N	To children out of parental control [BA2]	Leaf (infusion)	Oral
			Flu [BA1]	Leaf, fruit (see recipe 4)	Oral
*Euphorbia prostrata* Aiton (Euphorbiaceae) PCR 143	Sanguinho	N	Diarrhea [BA4]	Aerial (infusion)	Oral
*Foeniculum vulgare* Mill. (Apiaceae) PCR 14, 19, 35, 238, 239	Erva-doce	N	To calm down [BA2, PI2]	Aerial (infusion)	Oral
			Sore throat [BA2]	Aerial (infusion)	Oral
			Pain [BA3]	Aerial (infusion)	Oral
			Stimulate appetite [BA2]	Aerial (infusion)	Oral
			Gut motility due to food [BA1]	Aerial (see recipe 6)	Oral
			Liver [BA3]	Aerial (infusion)	Oral
			Flu [BA3]	Aerial (infusion)	Oral
			“Ventosidade” (flatulence) [BA1]	Aerial (see recipe 6)	Oral
			Cough [BA2]	Aerial (infusion)	Oral
*Gossypium hirsutum* L. (Malvaceae) PCR 24, 60, 118, 177	Algodão	N	Pain [BA1]	Fruit, flower (decoction, roast)	Topic
			Flu [BA1]	Leaf, fruit (see recipe 4)	Oral
			Symptoms of pneumonia [BA1]	Latex (added to recipe 4)	Oral
*Imperata brasiliensis* Trin. (Poaceae) PCR 169,198	Sapé	N	Baby teeth popping [BA1]	Leaf, root (infusion)	Oral, topic (bathing or tie the plant sprout to the child’s neck)
*Ipomoea batatas* (L.) Lam. (Convolvulaceae) PCR 243, 244	Batata-doce	E	Toothache [BA5]	Tuber (infusion)	Mouthwash
Iridaceae (undefined species) PCR 111	Cebola-branca	UNK	Labor (delivery of placenta) [BA1]	Tuber (see recipe 7)	Oral
*Kalanchoe pinnata* (Lam.) Pers. (Crassulaceae) PCR 222	Folha-da-costa, saião, folha-da-fortuna, among others	E	Flu [BA1]	Leaf (see recipe 8)	Oral
			Bronchitis [BA1]	Leaf (see recipe 8)	Oral
*Leonotis nepetifolia* L. R. Br. (Lamiaceae) PCR 99	Cordão-de-são-francisco	N	Gut [BA1]	Leaf (maceration)	Oral
			Fever [BA1]	Leaf (maceration)	Oral
			To remove blackmagic [BA1]	Leaf (maceration)	Topic (bathing)
*Lepidium bonariense* L. (Brassicaceae) PCR 332	Mastruz-rasteiro	N	Abdominal pain [BA3]	Aerial (infusion)	Oral
			Abdominal pain [BA1]	Aerial (maceration and add to milk)	Oral
*Lippia alba* (Mill.) N. E. Br (Verbenaceae) PCR 4, 13, 18, 33, 34, 62, 112, 195, 196, 325	Erva-cidreira, erva-cideira-roxa	N	To sleep [BA5]	Leaf (infusion)	Oral
			Hangover [BA1]	Aerial (see recipe 9)	Oral
			Hypertension [BA4]	Leaf (infusion)	Oral
			Abdominal bloating [BA4]	Leaf (infusion)	Oral
			Spiritual [BA2]	Aerial (see recipe 2)	Inhalation
*Lippia* sp. (Verbenaceae) PCR 167, 197	Erva-cidreira	UNK	Hypertension [BA4]	Leaf (infusion)	Oral
			Abdominal bloating [BA4]	Leaf (infusion)	Oral
			Sedative [BA2]	Aerial (infusion)	Oral
*Luffa cylindrica* M. Roem (Cucurbitaceae) PCR 333	Buchinha-do-norte, bucha	N	Abortive [BA2]	Fruit, seed (infusion)	Oral
			Sinusitis [PI2]	Fruit (infusion)	Inhalation
*Melinis minutiflora* P. Beauv. (Poaceae) PCR 225	Capim-gordura	N	Diabetes [BA5]	Leaf (infusion)	Oral
Menispermaceae (undefined species) PCR 178	Buticara, buti	UNK	Toothache [BA3, BA4]	Fruit (decoction)	Mouthwash
*Mentha arvensis* L. (Lamiaceae) PCR 20, 37, 183	Alevante, água-de-alevante, hortelã, hortelãzinho	E	Sedative [PI2]	Leaf (infusion)	Oral
			Measles [BA4]	Aerial (see recipe 10)	Oral
			Sexual weakness [PI2]	Leaf (infusion)	Oral
			Flu [BA4]	Leaf (syrup)	Oral
			Flu (fever, cough e body ache) [BA2]	Leaf (infusion)	Oral
			Labor (delivery of placenta) [BA1]	Aerial (see recipe 7)	Oral
*Mentha pulegium* L. (Lamiaceae) PCR 184	Puejo, poejo	E	Flu (fever, cough e body ache) [ba2]	Aerial (infusion)	Oral
			Flu [PI1]	Aerial (see recipe 11)	Oral
			Labor (delivery of placenta) [BA1]	Aerial (see recipe 7)	Oral
*Momordica charantia* L. (Cucurbitaceae) PCR 25, 97	Melão-de-são-caetano	E	Anemia [BA1]	Leaf (infusion or maceration)	Oral
			Itching [BA1]	Leaf (infusion)	Topic (bathing)
*Myracrodruon urundeuva* Allemão (Anacardiaceae) PCR 140	Aroeira	N	To bless [BA1]	Branches (in natura)	Topic
*Myristica fragans* Houtt. (Myristicaceae) PCR 114	Noz-moscada	E	“Quando comeu e ofendeu o intestino” (guts pain due to food)	Seed (see recipes 3 and 6)	Oral
			“Ventosidade” (flatulence) [BA1]	Seed (see recipe 6)	Oral
*Ocimum americanum* L. (Lamiaceae) PCR 229	Manjericão, alixir-paregórico	E	Flu [BA1]	Branches (see recipe 4)	Oral
*Ocimum basilicum* L. (Lamiaceae) PCR 21	Manjericão	E	Flu [BA1]	Branches (see recipe 4)	Oral
*Ocimum campechianum* Mill. (Lamiaceae) PCR 231, 232, 233	Alfavaca-fina, tiioiô, alfavaca	N	Flu (pediatric) [BA1]	Aerial (syrup)	Oral
			Wound healing [BA1]	Leaf (maceration)	Topic
*Ocimum gratissimum* L. (Lamiaceae) PCR 23, 230	Manjericão, tiioiô	E	Sedative [PI2]	Aerial (infusion)	Oral
			Flu [BA1]	Aerial (see recipe 4)	Oral
*Ocimum selloi* Benth. (Lamiaceae) PCR 5	Alixir-paregórico, elixirs	N	General pain [BA1]	Branches (infusion)	Oral
			Bowel pain [PI1]	Aerial (infusion)	Oral
*Operculina macrocarpa* (Linn) Urb. (Convolvulaceae) PCR 246	Batata-doce, batata-de-purga	N	Inflamed tooth, toothache [BA5]	Tuber (infusion)	Mouthwash
*Passiflora edulis* Sims. (Passifloraceae) PCR 38	Maracujá	N	Sedative [PI2]	Leaf (infusion)	Oral
*Persea americana* Mill. (Lauraceae) PCR 16, 247, 248	Abacate, abacate-branco	E	Weight loss [BA2]	Leaf (infusion)	Oral
			Leukemia [BA2]	Leaf (infusion)	Oral
			Cholesterol [BA5]	Seed (decoction)	Oral
			Liver [BA3]	Leaf (infusion)	Oral
			Kidney [BA3]	Leaf (infusion)	Oral
			Kidney stone [BA5]	Leaf (infusion)	Oral
*Petiveria alliacea* L. (Phytolaccaceae) PCR 117, 122, 256	Guiné	N	Pain due to dental caries (cavities) [BA1]	Root (thin pieces)	Topic
			Kidney [BA4]	Aerial (infusion)	Oral
			To prevent black magic [BA3]	Aerial (maceration in alcoholic drink)	Oral
			To prevent black magic [BA1]	Root (thin pieces)	Topic
*Phyllanthus amarus* Schumach. &Thonn. (Euphorbiaceae) PCR 147	Quebra-pedra	N	Inflammation [BA5]	Aerial (infusion)	Oral
			Kidney stone [BA3, BA4, BA5]	Aerial (infusion)	Oral
*Phyllanthus niruri* L. (Euphorbiaceae) PCR 146	Quebra-pedra	N	Inflammation [BA5]	Aerial (infusion)	Oral
			Kidney stone [BA3, BA4, BA5]	Aerial (infusion)	Oral
*Phyllanthus stipulatus* (Raf.) G. L. Webster (Euphorbiaceae) PCR 145	Quebra-pedra	N	Kidney stone [BA1]	Aerial (infusion)	Oral
*Phyllanthus tenellus* Roxb. (Euphorbiaceae) PCR 6	Quebra-pedra	N	Kidney stone [BA3, BA4, BA5]	Aerial (infusion)	Oral
			Inflammation [BA5]	Aerial (infusion)	Oral
*Plectranthus amboinicus* (Lour.) Spreng. (Lamiaceae) PCR 185, 323	Alfavaca-grossa, hortelã-grosso, hortelã-da-folha-grossa, hortelã-doce	E	Flu [BA1]	Leaf (see recipe 4)	Oral
			Flu [BA4]	Aerial (syrup)	Oral
			Expectorant [PI2]	Leaf (syrup)	Oral
			Eye inflammation [PI2]	Leaf (juice released when pressed with a hot plate)	Topic
			Gynecological infection [PI2]	Leaf (infusion)	Vulva and vagina bath using a bucket
*Plectranthus barbatus* Andrews (Lamiaceae) PCR 3, 29, 158	Boldo, boldo-grosso, folha-de-santa-bárbara	E	Indigestion [PI2]	Leaf (infusion)	Oral
			Stomach [BA1]	Leaf (infusion or maceration)	Oral
*Plectranthus neochilus* Schltr. (Lamiaceae) PCR 159, 160	Boldo, boldo-miúdo, boldo-unha-de-gato	E	Hangover [BA1]	Leaf (infusion)	Oral
			Gut [BA1]	Leaf (infusion)	Oral
			Indigestion [BA1, BA5]	Leaf (infusion)	Oral
*Pothomorphe umbellata* (L.) Miq. (Piperaceae) PCR 252	Capeba	N	Burning sensation on liver and stomach [BA1]	Leaf (infusion or maceration)	Oral
*Psidium* cf. *guajava* L. (Myrtaceae) [not collected]	Goiaba (guava)	N	Diarrhea [PI2]	Terminal portion of the branches (infusion)	Oral
*Punica granatum* L. (Punicaceae) PCR 216	Romã	E	Sore throat [BA1]	Bark, fruit, leaf (infusion)	Mouthwash
			Sore throat [PI1]	Bark, leaf (decoction)	Mouthwash
*Ricinus communis* L. (Euphorbiaceae) PCR 144	Mamona	E	Measles [BA4]	Seed (see recipe 10)	Oral
*Rosmarinus officinalis* L. (Lamiaceae) PCR 36, 186, 322	Alecrim	E	Body ache [BA2]	Branches (infusion)	Oral
			Flu [BA1]	Aerial (see recipe 4)	Oral
			To prevent black magic [BA2]	Branches (to bless)	Topic
			Whooping cough [BA2]	Branches (infusion)	Oral
*Ruta chalepensis* L. (Rutaceae) PCR 121, 148	Arruda-miúda	E	Otitis (pediatric) [BA1]	Aerial (maceration)	Topic
			Eye injury (knock) [BA1]	Aerial (juice)	Topic
			Flu [BA1]	Aerial (syrup)	Oral
			Against evil eye [BA1]	Aerial (in natura)	Put in a vase inside home
			Bronchitis [BA1]	Aerial (syrup)	Oral
*Ruta graveolens* L. (Rutaceae) PCR 123	Arruda	E	Baby belly button [BA2]	Aerial (oil extraction)	Topic
			Eye injury (knock) [BA1]	Aerial (juice)	Topic
			Otitis [BA1, BA2]	Aerial (maceration)	Topic
			Flu [BA1]	Aerial (syrup)	Oral
			To prevent black magic and envy [BA1]	Aerial (in natura)	Put in a vase inside home
			To prevent black magic and envy [BA2]	Aerial (fresh plant)	Topic
			Recovery and care after delivery [BA2]	Aerial (in natura)	Oral
			Bronchitis [BA1]	Aerial (syrup)	Oral
*Sambucus canadensis* L. (Caprifoliaceae) PCR 211, 212	Sabugueiro	E	Measles, chickenpox, smallpox [BA1, BA2]	Flower (infusion)	Oral or topic (bathing)
			Rheumatism [BA2]	Flower (maceration)	Topic
			Flu [BA1]	Leaf (syrup)	Oral
			Contusion [BA2]	Flower (maceration)	Topic
			Bronchitis [BA1]	Leaf (syrup)	Oral
*Sansevieria trifasciata* Prain (Liliaceae) PCR 58, 258	Espada-de-ogum, são-jorge	E	To remove black magic [BA2]	Leaf (incense)	Inhalation
*Schinus terebinthifolia* Raddi (Anacardiaceae) PCR 175	Aroeira	N	To bless [BA1]	Branches (in natura)	Topic
*Scleria distans* Poir. (Cyperaceae) PCR 132	Junço, dandá	N	Pain [BA1]	All parts (infusion)	Topic (bathing)
*Scoparia dulcis* L. (Scrophulariaceae) PCR 171	Bassorinha	E	Itching (pediatric) [BA1]	Aerial (infusion)	Topic (bathing)
			Bowel pain (pediatric) [BA1]	Aerial (infusion)	Oral
*Senna* sp. (Fabaceae) PCR 108	Copaíba	UNK	Hemorrhoid [BA1]	Stem (oil, resin extraction)	Topic
			Indigestion [BA1]	Stem (oil, resin extraction)	Oral
			Healing process [BA1]	Stem (oil, resin extraction)	Topic
*Sicana paineira* (Vell.) Naudin (Cucurbitaceae) PCR 334	Melão-coroá	E	Symptoms of heart attack [BA1]	Fruit (roast and grind)	Oral
*Sphagnetico latrilobata* (L.) Pruski (Asteraceae) PCR 156	Mal-me-quer	N	Menorrhagia (not related to pregnancy) [BA4]	Flower (infusion)	Oral
*Struthanthus marginatus* (Desr.) Blume (Loranthaceae) PCR 176	Erva-de-passarinho	N	Scabies (pediatric) [BA1]	Leaf (infusion)	Topic (bathing)
*Syzygium aromaticum* (L.) Merr. & L.M. Perry (Myrtaceae) PCR 124	Cravo-da-índia	E	Flu [BA1]	Flower (see recipe 4)	Oral
*Tithonia diversifolia* (Hemsl.) A. Gray (Asteraceae) PCR 154	Chapéu-de-couro	N	Urethra and kidney problems [BA1]	Aerial (infusion)	Oral
			Flu [BA1]	Aerial (infusion)	Oral
*Vernonia condensata* Baker (Asteraceae) PCR 157, 165	Boldo-do-chile, alumã	E	Hangover [BA1]	Leaf (see recipe 9)	Oral
			Indigestion [BA1]	Leaf (see recipe 9)	Oral
			Indigestion [BA4]	Leaf (infusion)	Oral
			Liver [BA4]	Leaf (infusion)	Oral
*Vernonia polyanthes* Less. (Asteraceae) PCR 8	Assa-peixe	N	Flu [BA1]	Leaf (syrup)	Oral
			For the lungs [BA1]	Leaf (maceration)	Oral
			Healing process [BA1]	Leaf (maceration)	Topic

^*^Native or naturalized (N), Exotic (E), Unknown origin (UNK)

**Table 3:** Recipes and medicinal plants from the origin place which uses were replaced by the informants after their migration to Bororé Peninsula. The plants were replaced by other species already known and with similar uses (see Table 2—maintenance) or by new species (Table Table 5 - incorporation)

Scientific name (family)/voucher	Popular names	Origin^*^	Popular use/[interviewee]	Plant part (preparation method)	Route
*Acacia adhaerens* Benth. (Fabaceae) PCR 82	Unha-de-gato	N	To bless [BA2]	Branches (in natura)	Topic
*Acosmium dasycarpum* (Vogel) Yakovlev (Fabaceae) PCR 131	Raiz-d’anta	N	Stomachache [BA2]	Bark (decoction)	Oral
*Ageratum conyzoides* L. (Asteraceae) PCR 27, 179	Mentrasto, mentrasto-branco	N	Dungal infection [BA5]	Aerial (topic, bathing)	Topic (bathing)
*Amburana cearensis* (Allemão) A.C. Sm. (Fabaceae) PCR 136, 138	Emburana	N	Headache [BA2]	Seed (see recipe 3)	Oral
			Indigestion [BA2]	Seed (toasting and brewing together with coffee)	Oral
*Anacardium* cf. *occidentale* L. (Anacardiaceae) [not collected]	Cajú (cashew)	N	Gut motility [BA2]	Fruit (in natura)	Oral
*Anadenanthera macrocarpa* (Benth.) Brenan (Fabaceae) PCR 79, 189	Angico, pau-barbado	N	Blood purifying [PI1, PI2]	Bark (decoction)	Oral
			Flu [PI1]	Bark (see recipe 12)	Oral
			Flu [PI2]	Bark (syrup)	Oral
Arecaceae (undefined species) PCR 193	Quitara	UNK	Flu [BA1]	Root (see recipe 8)	Oral
			Bronchitis [BA1]	Root (see recipe 8)	Oral
*Cestrum* sp. (Solanaceae) PCR 107, 170	Cuarana	UNK	To remove black magic [BA1]	Tuber (incense)	Inhalation
*Combretum* sp. (Combretaceae) PCR 92	Mufumbá	UNK	Inflammation [PI2]	Bark (soak in water, powder)	Topic
			Stop bleeding (injuries and scratches) [PI2]	Bark (soak in water, powder)	Topic
*Commiphora leptophloeos* (Mart.) J.B. Gillett (Burseraceae) PCR 220	Emburana, emburana-de-cambão, emburana-macho	N	Headache [BA2]	Seed (toast, grind and mix with oil)	Oral
			Indigestion [BA2]	Seed (toast, soak in water)	Oral
*Croton echioides* Müll. Arg. (Euphorbiaceae) PCR 268	Velame	N	Emetic [PI2]	Root (maceration, soak in water)	Oral
*Croton betulaster* Müll. Arg. (Euphorbiaceae) PCR 267	Pimentinha	N	To calm children down [BA2]	Root (infusion)	Oral
*Cymbopogon densiflorus* (Steud.) Stapf. (Poaceae) PCR 115	Capim-de-aruanda	N	To remove black magic [BA1]	Aerial (incense)	Inhalation
*Diptychandra aurantiaca* Tul (Fabaceae) PCR 204	Birro-branco	N	Emetic [PI2]	Bark (decoction)	Oral
Fabaceae (undefined species) PCR 129	Birro-cangalheiro, birro-branco	UNK	Emetic [PI2]	Root (maceration)	Oral
*Fevillea trilobata* L. (Cucurbitaceae) PCR 151	Gendiroba	N	Gut motility [BA1]	Seed, except pericarp (toast and add to coffee)	Oral
*Gallesia integrifolia* (Spreng.) Harms (Phytolaccaceae) PCR 217	Pau-d’alho	N	Pain [BA1]	Stem (decoction)	Topic (bathing)
			To remove black magic [BA1]	Stem (decoction)	Topic (bathing)
*Hymenaea courbaril* L. var. stilbocarpa (Hayne) Lee & Langenhein (Fabaceae) PCR 74, 130	Jatobá, jatobá-mirim	N	Flu [PI1]	Bark (see recipe 12)	Oral
*Hymenaea stigonocarpa* Mart. ex Hayne var. pubescens Benth. (Fabaceae) PCR 73, 78	Jatobá, jatobá-do-campo	N	Flu [PI1]	Bark (see recipe 12)	Oral
*Jacaranda puberula* Cham. (Bignoniaceae) PCR 67	Garobinha-do-mato, carobinha	N	Allergy [PI1]	Branches (infusion)	Topic (bathing)
*Jacaranda* sp. (Bignoniaceae) PCR 209, 210	Jacarandá	UNK	To bless [BA1]	Aerial (incense)	Topic
*Jatropha curcas* L. (Euphorbiaceae) PCR 259	Pinhão-branco, pinhão-manso	N	Skin burn [BA2]	Latex (ointment)	Topic
*Jatropha gossypiifolia* L. (Euphorbiaceae) PCR 261,262	Pinhão-roxo	E	Home protection [BA2]	Aerial (in natura)	–
*Julocroton fuscescens* (Spreng.) Baill. (Euphorbiaceae) PCR 59	Velame	N	Emetic [PI2]	Root (maceration)	Oral
*Lantana camara* L. (Verbenaceae) PCR 168	Cãmará	N	Healing process [BA1]	Leaf (maceration)	Topic
*Lecythis pisonis* Cambess (Lecythidaceae) PCR 120	Coco-de-sapucaia	N	Flu [BA1]	Fruit (see recipe 8)	Oral
			Bronchitis [BA1]	Fruit (see recipe 8)	Oral
*Myracrodruon urundeuva* Allemão (Anacardiaceae) PCR 140	Aroeira	N	Flu [PI1]	Bark (see recipe 12)	Oral
			Wash aggravated eyes [BA2]	Branches (infusion)	Topic
*Operculina macrocarpa* (Linn) Urb. (Convolvulaceae) PCR 246	Batata-doce, batata-de-purga	N	Blood purifying [PI2]	Tuber (grid and soak in water)	Oral
*Opuntia* sp. (Cactaceae) [not collected]	Palma	UNK	Labor (delivery of placenta) [BA1]	Branches (see recipe 7)	Oral
*Polygala* sp. (Polygalaceae) PCR 104, 163, 164	Cainaninha, puaia-branca	UNK	Flu [BA1]	Aerial (see recipe 8)	Oral
			Asthmatic bronchitis [BA1]	Aerial (see recipe 8)	Oral
*Ricinus communis* L. (Euphorbiaceae) PCR 144	Mamona	E	Laxative [BA5]	Seed (oil extraction)	Oral
			Wound [BA5]	Seed (oil extraction)	Oral
*Ruellia bahiensis* (Nees) Morong. (Acanthaceae) PCR 121, 141	Purga-do-campo	N	Fever [BA1]	All parts (infusion)	Oral
*Sanseviera cylindrica* Bojer (Liliaceae) PCR 242	Espada-de-ogum-fechada	E	To remove black magic [BA2]	Leaf (see recipe 2)	Inhalation
Sapotaceae (undefined species) PCR 109	Buranhê	UNK	Chronic wound [BA1]	Bark (decoction)	Topic
*Schinopsis brasiliensis* Engl. (Anacardiaceae) PCR 139	Braúna	N	Diarrhea [BA2]	Branches (infusion)	Oral
			To bless [BA2]	Branches (in natura)	Topic
*Senna spectabilis* (DC.) H.S. Irwin &Barneby (Fabaceae) PCR 84	São-joão	N	To bless [BA2]	Branches (in natura)	Topic
*Sida cordifolia* L. (Malvaceae) PCR 202	Malva-do-campo, malva-branca	N	Healing process [BA5]	Leaf(roast and grind)	Topic
*Sisyrinchium* sp. (Iridaceae) PCR 134	Capim-lanceta	UNK	Fever [BA2]	Leaf (infusion)	Oral
*Solanum americanum* Mill. (Solanaceae) PCR 101, 199, 250	Erva-de-santa-maria	N	Pneumonia [PI1]	Aerial (maceration)	Oral
*Ximenia americana* L. (Olacaceae) PCr 137	Ameixa-braba	N	Healing process [PI2]	Bark (maceration)	Topic

^*^Native or naturalized (N), Exotic (E), Unknown origin (UNK)

**Table 4:** Recipes and medicinal plants from the origin place which were no longer used by the informants (discontinuation) after their migration to Bororé Peninsula

Scientific name (family)/voucher	Popular names	Origin^*^	Popular use/[interviewee]	Plant part (preparation method)	Route
*Caesalpinia ferrea* Mart. Ex Tul. var. parvifolia (Fabaceae) PCR 125	Pau-ferro	N	Homemade mercury for medical use [BA5]	Bark (decoction)	Topic
*Chenopodium ambrosioides* L. (Chenopodiaceae) PCR 49, 61, 218, 253, 255, 328	Erva-de-santa-maria, mastruz, mentruz	N	Worm, vermifuge [PI1]	Aerial (see recipe 13)	Oral
			To wash an increased mosquito wound [PI1]	Aerial (maceration)	Topic
*Citrus* sp. (Rutaceae) [not collected]	Laranja (Orange)	E	“Sezão” (intermittent or cyclic fever, such as caused by malaria) [PI1]	Leaf (infusion)	Oral
*Diptychandra aurantiaca* Tul (Fabaceae) PCR 204	Birro-branco	N	Soap [PI2]	Bark (decoction)	Topic
Menispermaceae (undefined species) PCR 178	Buticara, buti	UNK	Paludism, typhoid fever [BA1]	Fruit (decoction)	Oral
*Operculina macrocarpa* (Linn) Urb. (Convolvulaceae) PCR 246	Batata-doce, batata-de-purga	N	Vermifuge [PI2]	Tuber (grid and soak in water)	Oral
*Ricinus communis* L. (Euphorbiaceae) PCR 144	Mamona	E	Worm, vermifuge [PI1]	Seed (see recipe 13)	Oral
*Ziziphus joazeiro* Mart. (Rhamnaceae) PCR 88	Juá	N	Dentifrice [PI2]	Bark (maceration)	Topic
			Dandruff shampoo [PI2]	Bark (decoction)	Topic

^*^Native or naturalized (N), Exotic (E), Unknown origin (UNK)

**Table 5:** Recipes and medicinal plants which uses were incorporated at the informants’ therapeutic after their migration to Bororé Peninsula

Scientific name (family)/voucher	Popular names	Origin^*^	Popular use/[interviewee]	Plant part (preparation method)	Route
*Achillea millefolium* L. (Asteraceae) PCR 10	Novalgina	E	Headache [PI1]	Aerial (infusion)	Oral
*Aesculus hippocastanum* L. (Hippocastanaceae)^**^	Castanha-da-índia	E	Varicose veins [BA5]	Seed (see recipe 14)	Oral
*Aloysia gratissima* (Gillies & Hook.) Tronc. (Verbenaceae) PCR 17	Alfazema, fazema	N	Stress [BA2]	Branches (in natura)	Throw over the head
*Aloysia triphylla* Royle (Verbenaceae) PCR 40	Erva-cidreira, cidreira	E	Sedative [PI1]	Aerial (infusion)	Oral
*Bacharis trimera* (Less.) DC. (Asteraceae) PCR 50, 181	Carqueja, carqueja-de-praia	N	Stomach [BA5]	Leaf (infusion or maceration)	Oral
*Bidens pilosa* L. (Asteraceae) PCR 2, 71, 153, 180	Picão	N	Hepatitis [BA4]	Aerial (see recipe 15)	Topic (bathing)
			Fungal infection [BA5]	Aerial (topic, bathing)	Topic (bathing)
*Brassica* cf. *oleraceae* L. (Brassicaceae) [not collected]	Couve (cabbage)	E	Gastritis [PI1]	Leaf and stem (juice)	Oral
*Calea pinnatifida* (R. Br.) Less. (Asteraceae) PCR 47	Cipó-cruz	N	Pain [BA3]	Aerial (infusion)	Oral
			Liver [BA3]	Aerial (infusion)	Oral
			Stomach [BA5]	Aerial (maceration)	Oral
			Kidney [BA3]	Aerial (infusion)	Oral
			to remove black magic [BA3]	Aerial (infusion)	Topic (bathing)
*Cinnamomum* sp. (Lauraceae) [not collected]	Canela (cinnamon)	E	Flu [BA4]	Bark (syrup)	Oral
*Cissus verticilata* (L.) Nicholson & CE Jarvis (Vitaceae) PCR 11, 45, 55	Insulina	N	Diabetes [BA1, BA2]	Leaf (infusion)	Oral
*Eucalyptus* sp. (Myrtaceae) PCR 203	Eucalipto, eucalipto-verdadeiro	E	Inhaler [BA5]	Leaf (infusion)	Inhalation
*Galium hypocarpium* (L.) Endl. Ex. Griseb. (Rubiaceae) PCR 65	Erva-de-bicho	N	Hepatitis [BA4]	Aerial (see recipe 15)	Topic (bathing)
*Ginkgo biloba* L. (Ginkgoaceae)^**^	Ginkgo biloba	E	Varicose veins [BA5]	Leaf (see recipe 14)	Oral
*Imperata brasiliensis* Trin. (Poaceae) PCR 169,198	Sapé	N	Diabetes [BA5]	Leaf (infusion)	Oral
*Lactuca* cf. *sativa* L. (Asteraceae) [not collected]	Alface	E	Sedative, to sleep [PI1]	Stem (infusion)	Oral
*Lippia alba* (Mill.) N. E. Br (Verbenaceae) PCR 4, 13, 18, 33, 34, 62, 112, 195, 196, 325	Erva-cidreira, erva-cideira-roxa	N	Sedative [PI1, PI2]	Aerial (infusion)	Oral
*Matricaria* cf. *chamomilla* L. (Asteraceae) [not collected]	Camomila (chamomile)	E	Sedative [PI1]	Flower, leaf (infusion)	Oral
*Melissa officinalis* L. (Lamiaceae) PCR 31	Erva-cidreira, melissa	E	Sedative [PI1, PI2]	Aerial (infusion)	Oral
*Mentha citrata* Ehrn. (Lamiaceae) PCR 226	Hortelã	N	Vermifuge [PI2]	Leaf (see recipe 1)	Oral
*Mentha pulegium* L. (Lamiaceae) PCR 184	Puejo, poejo	E	Flu [BA4]	Aerial (infusion or syrup)	Oral
*Mikania glomerata* Spreng. (Asteraceae) PCR 52	Guaco	N	Flu [BA4]	Aerial (syrup)	Oral
			Flu [PI1]	Aerial (infusion)	Oral
			Flu [BA1]	Aerial (see recipe 4)	Oral
			Flu [PI1]	Aerial (see recipe 11)	Oral
*Musa* sp. (Musaceae) [not collected]	Banana	UNK	Headache [BA2]	Leaf (infusion)	Oral
			Vitamin [BA2]	Fruit (in natura)	Oral
			Bruised bone [BA2]	Leaf, latex (maceration)	Topic
*Ocimum campechianum* Mill. (Lamiaceae) PCR 231, 232, 233	Alfavaca-fina, tiioiô, alfavaca	N	Indigestion [PI1]	Leaf (infusion)	Oral
*Opuntia* sp*.* (Cactaceae) PCR 56	Palma-japonesa	UNK	Mental illness affecting humor [BA2]	Branches (infusion)	Oral
*Phyllanthus niruri* L. (Euphorbiaceae) PCR 146	Quebra-pedra	N	Regulate urination [PI2]	Aerial (infusion)	Oral
*Phyllanthus tenellus* Roxb. (Euphorbiaceae) PCR 6	Quebra-pedra	N	Regulate urination [PI2]	Aerial (infusion)	Oral
*Plantago major* L. (Plantaginaceae) PCR 201	Transagem	E	Uterus inflammation [BA5]	Leaf (infusion)	Vulva and vagina bath using a bucket
*Plectranthus barbatus* Andrews (Lamiaceae) PCR 3, 29, 158	Boldo, boldo-grosso, folha-de-santa-bárbara	E	Coffee addiction [PI2]	Leaf (infusion)	Oral
*Rosmarinus officinalis* L. (Lamiaceae) PCR 36, 186, 322	Alecrim	E	Enlarged heart (cardiomegaly) [PI2]	Branches (infusion)	Oral
*Senna* sp. (Fabaceae) PCR 108	Copaíba	UNK	Hemorrhoid [PI1]	Stem (oil, resin extraction)	Topic
*Vernonia polyanthes* Less. (Asteraceae) PCR 8	Assa-peixe	N	Dandruff and seborrhea [PI2]	Branches (infusion)	Topic
*Zingiber* cf. *officinale* Roscoe (Zingiberaceae) [not collected]	Gengibre (ginger)	E	Hoarseness [PI1]	Root (in natura, infusion)	Oral
			Cough [PI1]	Root (in natura, infusion)	Oral

^*^Native or naturalized (N), Exotic (E), Unknown origin (UNK)

^**^herbal product purchased in the market

The informants also mentioned 15 recipes containing two or more plants and informed when these recipes were changed after migration (Table 6). These recipes were not unchangeable, since they stated that some plants could be included or replaced keeping the formula similarly effective.

**Table 6:** Recipes containing two or more species and their methods of preparation

Recipe number	Original recipe	Current recipe (after migration)
1	Infusion with garlic (*Allium sativum*)	Infusion of garlic bulb (*A. sativum*) and mint leaves (*Mentha citrata*)
2	Incense prepared with leaves of *Sanseviera cylindrica*, leaves of *Aloysia gratíssima* and *Lippia alba*	*Sanseviera cylindrica* replaced by species with similar use, when possible
3	Decoction of roasted seeds of *Amburana cearenses* and seeds of *Myristica fragans* together with coffee (*Coffea arabica*)	Recipe still used for gut pain, but the use of *A. cearenses* for headache was replaced
4	Syrup prepared with cambuci (not collected), barks of pineaple (*Ananas comosus*), leaves of *Cymbopogon citratus*, leaves or fruits of *Gossypium hirsutum*, leaves of *Eugenia uniflora*, aerial parts of *Rosmarinus officinalis*, branches of manjericão (*Ocimum americanum* or *Ocimum basilicum*), leaves of *Plectranthus amboinicus*, and flowers of *Syzygium aromaticum*	Same recipe, but aerial part of *Mikania glomerata* was incorporated
5	Leaves of lime and orange (*Citrus sp*.) macerated and mixed with creolin	The same
6	Decoction prepared with seeds of *Myristica fragans* and aerial parts of *Foeniculum vulgare*	The same
7	Infusion of aerial parts of *Mentha arvensis*, branches of *Opuntia sp.* (Cactaceae), aerial parts of *Mentha pulegium*, and tuber of cebola-branca (undefined species, Iridaceae)	*Opuntia sp*. is no longer used in the recipe
8	Syrup containing, fruits of *Lecythis pisonis*, aerial parts of *Polygala sp*. (Polygalaceae), roots of quitara (undefined species, Arecaceae), leaves of *Kalanchoe pinnata* and garapia (not collected)	*Lecythis pisonis*, *Polygala sp*. and quitara (Arecaceae) replaced by plants with similar uses, like garlic (*Allium sativum*)
9	Infusion of leaves of *Vernonia condensata* and aerial parts of *Lippia alba*	The same
10	Infusion of aerial parts of *Mentha arvensis* and seed oil of *Ricinus communis*	The same
11	Infusion of aerial parts of *Mentha pulegium*	Syrup prepared with aerial parts of *Mentha pulegium* and *Mikania glomerata*
12	Syrup prepared with barks of *Anadenanthera macrocarpa*, barks of *Myracrodruon urundeuva* and jatobá (*Hymenaea courbaril* or *Hymenaea stigonocarpa*)	Recipe replaced by the use of single species
13	Maceration prepared with saffron (not collected), aerial parts of *Chenopodium ambrosioides* and mamona oil (*Ricinus communis*)	Recipe discontinued
14	None	Herbal remedy containing seeds of *Aesculus hippocastanum* and leaves of *Ginkgo biloba*
15	None	Infusion of aerial parts of *Bidens pilosa* and aerial parts of *Galium hypocarpium*

### Therapeutic categories and informant’s consensus factor

The popular uses cited by the participants were grouped in 15 therapeutic classes (Table 7). The most relevant categories of use in our sample were respiratory (62 citations), gastrointestinal (58 recipes) and inflammation, pain and fever (55 citations). A similar study with people who migrated from Northeastern Brazil to Diadema (also a metropolitan region of São Paulo) found the same categories as the most cited [18]. In fact, studies with urban population have shown that the use of medicinal plants is more common for treatment of “minor” problems, like gastritis, cough, and contusion, if compared to “serious” diseases like cancer, psychiatric disorders, and neurological conditions [3, 30, 31].

**Table 7:** Popular uses, number of species used (*N*_t_), number of use citations (recipes) (*N*_ur_), and informant’s consensus factor (ICF) calculated for each therapeutic category

Therapeutic category	Popular uses	*N*_t_	*N*_ur_	ICF
Birth control, childbirth	Abortive and post-abortion care, labor (delivery of the placenta), childbirth, for the baby to be born faster, recovery, and care after delivery	8	9	0.13
Cardiovascular and hematological	To the heart, hypertension, symptoms of heart attack, enlarged heart (cardiomegaly), blood purifying, varicose veins, leukemia	10	11	0.10
Contagious and tropical diseases	Measles, chickenpox, smallpox, paludism, typhoid fever, “sezão” (intermittent or cyclic fever, such as caused by malaria)	5	6	0.20
Dermatological	Allergy, itching, scabies, fungal infection, healing process, skin burn, wound, chronic wound, to wash an increased mosquito wound, “bicheira” (pests, as *Tunga penetrans*), homemade mercury for medical use, stop external bleeding (injuries and scratches)	21	23	0.09
Endocrine and metabolism	Diabetes, cholesterol	4	5	0.25
Gastrointestinal	To the stomach, liver, gut, indigestion, hangover, burning sensation on liver and stomach, gastritis, hepatitis, emetic, gut motility, guts pain due to food, worm, vermifuge, hemorrhoid, laxative, diarrhea, “ventosidade” (flatulence)	34	58	0.42
Genitourinary	Kidney, kidney stone, urethra and kidney problems, infection with vaginal discharge, difficulty urinating, regulate urination, gynecological infection, uterus inflammation, menorrhagia	14	23	0.41
Hygiene	Domestic hygiene, to vent the house, soap, dentifrice, dandruff shampoo, armpit odor, seborrhea	6	7	0.17
Inflammation, pain and fever	Pain, fever, inflammation, body ache, headache, inflamed tooth, toothache, pain due to dental caries (cavities), sore throat, inflammation from inside the body, feeling sick, contusion, tendon inflammation, bruised bone, bowel pain, abdominal pain, abdominal bloating, eye pain, eye injury (knock), eye inflammation, wash aggravated eyes, sinusitis, otitis, rheumatism, hoarseness	40	55	0.20
Magical	Spiritual, to remove black magic, against evil eye, to bless, to prevent envy, home protection, against stress (external use)	21	26	0.20
Neonatal care	Baby belly button, baby teeth popping	3	3	0.00
Psychoanaleptic	Sedative, to sleep, to calm down, to children out of parental control, bad feeling, mental illness affecting humor	15	24	0.39
Respiratory problems	Flu, cold, cough, whooping cough, breast congestion, expectorant, inhaler, catarrh, bronchitis, asthmatic bronchitis, pneumonia, for the lungs	38	62	0.39
Stimulant and fortifier	Anemia, stimulate appetite, vitamin, sexual weakness	4	5	0.25
Others	Coffee addiction, weight loss	2	2	0.00

The therapeutic categories with higher ICF were gastrointestinal (0.42), genitourinary (0.41), psychoanaleptic (0.39), and respiratory (0.39), while the other categories presented ICF lower than 0.25 (Table 7). Low ICF values indicate that plants are chosen randomly or the informants do not exchange information about their use [28, 29]. As we can see, there was a low consensus among the participants, which in many cases cited different plants and uses for similar ailments. The most accepted interpretation is that the migrants do not share information about the species and uses, possibly because in many cases, the access to medicinal plants is no longer the primary health care adopted. Another hypothesis is that the adaptation to a new environment, with access to different medicinal plants, resulted in a heterogeneous use among the migrants, which reflects the low ICF value found for most therapeutic categories. However, when we analyze the species and indication cited by migrants from the same state and biome, we can observe higher agreement of use (see Tables 2, 3, 4, and 5) suggesting that migrants that share a common background are more likely to exchange information. Low ICV values were also found in a previous study that evaluated the dynamics of use of medicinal plants among migrants living in Diadema [18].

Table 8 shows the 19 species cited at least five times by the interviewees. The species most cited were *Foeniculum vulgare* (10 recipes from 4 informants), *Baccharis trimera* (9 recipes from 3 informants), and *Ruta graveolens* (9 recipes from 2 informants)—see Tables 2, 3, 4, and 5. Table 8 also shows the index of relative importance of each plant, represented by the number of informants that cited the plant proportionally to the number of recipes. *Lippia alba* was the species used by the highest number of informants, with a relative importance of 0.86, although cited to five different indications. A high relative importance (0.80) was also observed to *Phyllanthus niruri* and *Phyllanthus tenellus* (used to treat kidney stones by three migrants). On the other hand, we observed that *Ruta chalepensis* and *Ruta graveolens* are used by few informants for many different purposes. This suggests that these species may have particular importance for these informants, but not for the other migrants. It is interesting to note that all species in this list had the use maintained after migration, except for *Calea pinnatifida*, which use was incorporated by two informants after their migration to the Bororé Peninsula.

**Table 8:** Index of relative importance and dynamic of use of the most cited species

Species	Number of citations	Number of informants	Dynamics^*^	Index^**^
*Aloysia gratissima*	5	2	M, I	0.40
*Anadenanthera macrocarpa*	6	3	M, R	0.50
*Bacharis trimera*	9	3	M, I	0.33
*Cajanus cajan*	8	4	M	0.50
*Calea pinnatifida*	5	2	I	0.40
*Chenopodium ambrosioides*	7	3	M, D	0.43
*Cymbopogon citratus*	8	4	M	0.50
*Foeniculum vulgare*	10	4	M	0.40
*Lippia alba*	7	6	M, I	0.86
*Mentha arvensis*	6	4	M	0.66
*Mentha pulegium*	6	4	M, I	0.66
*Persea americana*	6	3	M	0.50
*Phyllanthus niruri*	5	4	M, I	0.80
*Phyllanthus tenellus*	5	4	M, I	0.80
*Plectranthus amboinicus*	5	3	M	0.60
*Rosmarinus officinalis*	5	3	M, I	0.60
*Ruta chalepensis*	5	1	M	0.20
*Ruta graveolens*	9	2	M	0.22
*Sambucus canadensis*	6	2	M	0.33

^*^M = maintained; I = incorporated; D = discontinued

^**^Index of relative importance = number of informants/number of citations

### Dynamics of use

The category where each species was classified (maintenance, replacement, discontinuation, and incorporation) was defined according to the dynamics of use described by the interviewee after his/her migration to the Bororé Peninsula. One species could be used for different purposes (more than one recipe) and the dynamics of use could be different for each recipe and informant.

Table 9 shows the number of recipes and percentage of maintenance, replacement, incorporation, and discontinuation for each recipe considering each informant and the total sample. We observed that, on average, most uses were maintained (65.4%) after the migration (206 recipes containing 80 species), 54 recipes (17.1%) containing 39 species were replaced by plants available in the Bororé Peninsula, 45 new recipes (14.3%) were incorporated, and only 3.2% fell into disuse after migration, but these percentages are very different if we analyze the dynamic of use for each informant. It is clear that the knowledge about medicinal plants is very different among the participants. BA1 and BA2 cited most plants and recipes while BA3 showed a limited use of medicinal plants. It was previously reported that a large part of the knowledge about medicinal plants is not shared among migrants, and in many cases, the same species are used differently or for different ailments [13].

**Table 9:** Number of species and recipes cited by the informants and their dynamic of use

Migrant	Number of species	Number of recipes	Recipes/species	Dynamic of use: number of recipes (percentage)			
				Maintained	Replaced	Discontinued	Incorporated
BA1^*^	70	110	1.57	91 (82.7)	16 (14.5)	1 (0.9)	2 (1.8)
BA2^**^	34	59	1.74	37 (62.7)	16 (27.1)	0 (0.0)	6 (10.2)
BA3^*^	11	19	1.72	15 (78.9)	0 (0.0)	0 (0.0)	4 (21.1)
BA4^*^	20	28	1.40	23 (82.1)	0 (0.0)	0 (0.0)	5 (17.9)
BA5^*^	26	27	1.04	14 (51.9)	5 (18.5)	1 (3.7)	7 (25.9)
PI1^**^	25	37	1.48	11 (29.7)	8 (21.6)	4 (10.8)	14 (37.8)
PI2^**^	29	35	1.21	15 (42.9)	9 (25.7)	4 (11.4)	7 (20.0)
Total	131	315	2.40	206 (65.4)	54 (17.1)	10 (3.2)	45 (14.3)

^*^migrants from Atlantic Forest biome

^**^migrants from Caatinga biome

**Fig. 3:**
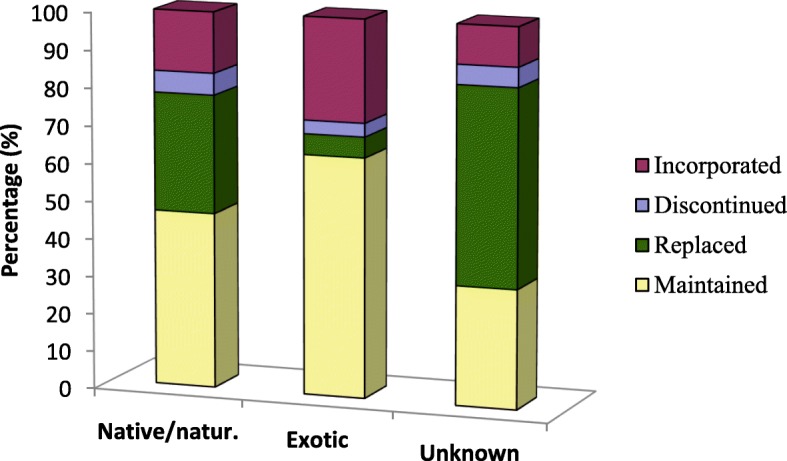
Percentage of species native/naturalized, exotic, or from unknown origin which were maintained, replaced, discontinued, or incorporated

In general, we could observe highest rates of maintenance with migrants from Atlantic Forest (especially BA1, BA3, and BA4), while migrants from Caatinga biome (BA2, PI1, and PI2) presented lower percentages of maintenance (compared with the average of the total sample) and showed higher rates of replacement, incorporation, and discontinuation (Table 9). This data suggest that native species from Caatinga biome were not available in the host place (Atlantic Forest biome) and could not be easily cultivated or acquired there. In fact, when we compared the species identified as native/naturalized or exotic, the percentage of maintained uses was found to be higher for exotic species, while the replacement and discontinuation was higher for native species (Fig. 3).

These dynamics of use and importance of therapeutic categories were previously discussed by several authors. Medeiros et at [1, 19] reported that migrants can adopt two main strategies when arriving the new environment: adaptation of the ethnomedical system to the new flora of the new place and acquisition of the original plants from the original place. The authors did not focus on internal migrations within a country, but many variables discussed are similarly valid in our study. Among the possible adaptations discussed, they cite the incorporation of new plant species or new uses for known species in the migrant pharmacopeia and the replacement of plants from the original flora by species with phylogenetic proximity or that possess similar morphological, chemical, and sensory characteristics. Leonti [32] also discusses the displacement of people and cross-cultural knowledge exchange using different concepts. The author reports that cultural interaction may alter the diversity and the importance of medicinal plants, which is detectable as continuity and disjunction or discontinuity and synchronism [32, 33].

It is important to point out that the study with migrant people has some limitations. In our study, we intended to compare the current use of medicinal plants by migrants living in a metropolitan region and the therapeutic resource they had before migrating. However, we could not measure the “latent knowledge,” which means that some species that were used in their hometowns possibly were not remembered, because they were not found in the host place or their use (indication) was no longer necessary. In this case, the number of replaced and discontinued species and recipes found in our study is possibly underestimated. Other factors can affect the chance of plants being forgotten, such as changes in the importance of certain use categories and differences in the prevalence of diseases [32]. In our study, most of the abandonments occurred for species used as vermifuges and to treat contagious or tropical diseases; the informants related that these problems could be treated with cheap and effective allopathic medicines (vermifuges) or the diseases are uncommon in the new environment (tropical diseases). Some authors warn that it is difficult to estimate how much knowledge about medicinal plants has been lost by migrants in comparison with people from their place of origin because, in most cases, no baseline data exist [1]. We carried out a bibliographic search in scientific databases and no ethnobotanical studies were found for the six cities of origin of our participants.

Another point for consideration is the importance of certain therapeutic resources which uses are maintained by migrants, despite their displacement to regions with different biome and culture and with better access to healthcare facilities. It was not our objective to search in databases if the popular use reported by the participants was already proven by scientific studies. However, we can observe that several plants cited were evaluated in pre-clinical studies or recognized by its traditional use, but only a few species were licensed as an herbal drug in the Brazilian market. As an example, we can cite *Aesculus hippocastanum* (antivaricose), *Ananas comosus*, *Eucalyptus* sp. and *Mikania glomerata* (expectorant), and *Matricaria chamomilla* (anxiolytic) which popular use is related to the therapeutic category approved [34]. In other cases, the species is licensed as an herbal product in a therapeutic category, but different popular uses are cited by the informants: *Alpinia zerumbet* (cited for cardiovascular use and registered as antispasmodic), *Caesalpinia ferrea* (employed as homemade mercury and approved as expectorant), *Ginkgo biloba* (used for varicose vein and approved as antivertiginous, antiplatelet agent and vasodilator), *Lantana camara* (cited as healing and registered as expectorant agent), *Persea americana* (several popular uses, but different of the registered use—anti-inflammatory), *Schinus terebinthifolius* (magical use and registered as anti-infective and healing agent), *Syzygium aromaticum* (used to treat flu and licensed as anti-dyspeptic drug), and *Zingiber officinale* (used to treat cough and hoarseness, but registered as antiemetic and antinauseant).

### Qualitative analysis—the dynamics of medicinal plants use

As previously discussed by other authors, many factors can affect the use of medicinal plants by migrants: differences in the flora of the new environment, the access to cultivated or fresh medicinal plants, the local culture and knowledge about medicinal plants, prevalence of diseases and access to health system, media influence, among others [1, 10, 13, 15, 18, 35]. The reasons that drive the dynamics of medicinal plant use by different migrants may also depend on personal choices. The qualitative analysis of the interviews allows understanding the individual reasons that justify the maintenance, replacement, abandonment, or incorporation of certain medicinal plant uses.

The migrants interviewed stated that upon their arrival in São Paulo, they looked for plants previously known in their hometowns. Most medicinal uses of species already known in their original regions were maintained at Bororé Peninsula, mainly for exotic plants or species with wide geographical distribution, as previously discussed. In many cases, native species from the Atlantic Forest (predominant biome of Esplanada, Jitaúna, and Itabuna) were also found at Bororé. On the other hand, the maintenance of species endemic to the Caatinga was difficult due to the considerable climatic difference, which made it challenging to find these species or to cultivate them in the new biome at São Paulo metropolitan region.

In some cases, we observed the cultivation of plants they considered of greater importance. For instance, BA1 received from his nephew (coming from Bahia) seeds and seedlings of *Amburana cearensis* and *Commiphora leptophloeos*, both species known as emburana and employed to alleviate the symptoms of indigestion. The migrants also used to find the same species of their original regions in local emporiums, herbal houses, or street fairs, as described by other authors [36–38]. However, the interviewees prefer to avoid getting plants from these sources whenever possible, because the botanical material is generally kept in bad conditions and subjected to contamination and deterioration, as also described in other studies [39, 40].

When the migrants did not find the species from their original region, they tried to replace them with similar plants from the new region guided by different strategies: conversation with neighbors; information from popular books and local media; and observing the ingestion of plants by animals or looking for species with organoleptic characteristics similar to those of the original species, in order to reach similar effects, as also observed with other populations [18, 41–43]. In some cases, several plants were mentioned as possible options (substitutes) for the replaced plants and recipes.

In our study, we observed that many species of the same genus, although recognized as different plants by the participants, were employed for the same indications: *Ocimum americanum*, *O. basiicum*, *O. campechianum*, and *O. gratissimum* (employed to treat flu) or *Phyllanthus amarus*, *P. niruri*, and *P. tenellus* (used as anti-inflammatory and against kidney stone). We also observed that even species from different genus and family, but sharing similar morphology or characteristics like odor and taste, are used for similar purposes, as is the case of *Aloysia triphylla* and *Lippia alba* (Verbenaceae) both known as erva-cidreira and used as a sedative/to sleep or *Plectranthus barbatus* (Lamiaceae) and *Vernonia condensata* (Asteraceae) known as boldo/boldo-do-chile and employed for hangover and indigestion. This makes sense considering that plants with close organoleptic properties have higher chance to have similar chemical constitution and that morphological and organoleptic properties are the basis for the doctrine of signature [32, 41, 44].

As previously mentioned, many species not found were from Caatinga biome and were replaced by local ones. Examples of species replaced are pimentinha (*Croton betulaster*), employed as anxiolytic/sedative in Novo Horizonte and replaced by pitanga (*Eugenia uniflora*) or são-joão (*Senna spectabilis*) and braúna (*Schinopsis brasiliensis*), employed as magical (to bless) by relatives of BA2 and replaced by alfazema (*Aloysia gratissima*). At the same time that some native species were replaced, in particular those endemic, other naturalized or exotic plants were incorporated. This fact can be explained by the increasing cultivation and adaptation of several exotic medicinal plants in different geographic regions, as observed to sálvia (*Salvia officinalis*) and camomila (*Matricaria chamomilla*) and by the use of teas and sachets infusions from Asian and European species, which are easily found in supermarkets, as reported by other studies carried out in different Brazilian regions [45–47].

In addition, our study suggests that the introduction of species from Bororé’s Atlantic Forest on the migrants’ therapeutic resources is slower than the introduction of exotic species often cultivated in the city of São Paulo. Moreover, some species that are currently found in the entire country were incorporated into the interviewees’ therapeutic practice only after they moved to Bororé Peninsula, as is the case of guaco (*Mikania glomerata*).

Occasionally, the migrants’ therapeutics would include different species for the same purpose and they could maintain the use when one or more species were found in Bororé. Interviewee BA1 used both the capim-de-aruanda (*Cymbopogon densiflorus*) and guiné (*Petiveria alliacea*) to prevent black magic, but since he could not find the first species in Bororé Peninsula, he limited himself to the second one. On the other hand, we observed that the knowledge about the occurrence and medicinal properties of some plants from Bororé was not always shared among the residents. Interviewees BA3 and BA4 alleged to maintain the use of buticara (Menispermaceae—undetermined species) for toothache, collecting the plant on Bororé’s Forest, while BA1 did not find this species. Similarly, interviewee BA2 maintained the use of angico (*Anadenanthera macrocarpa*) as anxiolytic (“when you have a bad feeling”) and for wound treatment, while PI1 and PI2 did not find the species and discontinued its use against flu, cold, and for cardiovascular problems (blood purifying). BA1 reported to find aroeira (*Myracrodruon urundeuva*) at Bororé’s Forest and maintained its use to bless (magical), while PI1 did not find the species on the local Forest and changed the recipe (formula) used to prepare an expectorant syrup to treat flu and cold. The same strategies of replacement and incorporation were cited by Garcia et al. [18] for a similar group of migrants.

Vegetables and fruits often found in the migrants’ diet with attributed medicinal properties were also named. Some vegetables were already used in their hometown in either or both contexts (as food and medicine), such as lettuce (*Lactuca sativa*) and pomegranate (*Punica granatum*), employed by PI1 as a sedative and to treat sore throat, respectively. Other examples are pineapple (*Ananas comosus*), pitanga (*Eugenia uniflora*), and basil (*Ocimum basilicum*), employed against flu by BA1. The diet of the interviewees was altered in order to consume more vegetables classified as prophylactic or useful for the treatment of diseases acquired or detected in the new environment, as also reported in other studies [4, 5, 14, 48, 49]. An increase in knowledge about food medicines was also observed in migrants from the Dominican Republic living in New York [20], in agreement with our data pointing that the acquisition of vegetables (including medicinal food) in big cities may be facilitated.

When we consider the category of use, we observe that several incorporations are related to pathologies that the informants claimed do not exist or be very uncommon in their hometowns. Plants reported to act as sedatives such as chamomile (*Matricaria chamomilla*) and erva-cidreira (*Aloysia triphylla* and *Lippia alba*) began to be used against stress because the city life imposes a greater risk to mental health, according to interviewees. However, it is likely that some diseases could not be diagnosed in their hometowns, because some diagnostics would require sophisticated laboratory tests and, at the time they moved, their hometowns did not have an adequate public health system. As an example, we can cite the cardiac hypertrophy detected in PI2 by clinical exams performed after relocation to a metropolitan area and treated with rosemary (*Rosmarinus officinalis*).

Several discontinuations occurred due to the availability of alternative therapies or allopathic medicines with low cost, as the use of mentruz (*Chenopodium ambrosioides*), castor oil extracted from castor beans (*Ricinus communis*), and batata-de-purga (*Operculina macrocarpa*) against worms, which were less palatable than allopathic medicines according to the interviewees, and because these medications are sold over the counter. In addition, access to basic sanitation also contributed to the decreased incidence of infectious diseases.

Other plants were no longer used for some purposes (discontinuation/abandonment), and industrialized products were used in their place. As an example, we can cite pau-ferro (*Caesalpinia ferrea*), used by BA5 on the preparation of home mercury, or plants used to make products of personal hygiene, as juá (*Ziziphus joazeiro*), which bark was used by PI1 to get a dentifrice and an anti-dandruff shampoo, or birro-branco (*Diptychandra aurantiaca*), used by PI2 to prepare home soap in his hometown. The use of industrialized products instead of natural products may be explained possibly because the access to drugstores and industrialized products is easier in the urban metropolis, and also, due to the best economic situation and purchasing power of the migrants in relation to the time when they lived in their hometowns. According to Haselmair et al. [50], the continuation of the traditional medicinal health practices is challenged by increasing industrialization and globalization where the use of medicinal plants is starting to play a secondary role.

Even though the interviewees had better access to drugstores, they kept the use of several medicinal plants and named some species with brand names of allopathic drugs, which suggests that the use of medicinal plants by the informants is not falling away, but being constantly modified. We can cite as an example the Novalgina (*Achillea millefolium*), employed as analgesic by PI1, and the insulin (*Cissus verticillata*) used by BA1 and BA2 to reduce the hyperglycemia. In fact, the use of active principles or brand name of drugs to name medicinal plants was previously related in other studies [18, 51].

Taken together, these reports are in agreement with previous literature showing that the use of medicinal plants in an urban context is not static and is constantly changing and adapting to the current life conditions [1, 2, 19, 32]. It is important to highlight that the seven informants, despite characterized as experts and users of medicinal plants, began to use the public health service system, since they considered the official therapeutics an additional treatment option. Access to the public system also influenced the discontinuation of plants to treat some infectious diseases, since they had access to vaccination programs. However, the interviewees declared that they kept the use of medicinal plants whenever their experiences indicated that it would be more efficacious than pharmaceutical drugs. Other studies [10, 36, 52, 53] also observed the simultaneous use of traditional and official therapeutic as a consequence of living in an urban region with easy access to drugstores and public health. In a study with Asian migrants living in the UK, up to 82% of participants who took prescription medicines did not tell their healthcare professionals about any herbal medicine they consumed [9]. Health professionals should be aware of this concomitant treatment option, since it can alter the efficacy and safety of many drugs [38, 54, 55].

## Conclusion

We observed cross-cultural adaptations on the migrants’ ethnomedicine after migration to a metropolitan region. Factors like the biome and occurrence of the species, prevalence of some diseases, and the local knowledge were listed as reasons to change the use of medicinal plants. The migration extended their knowledge regarding the diversity of therapies available in a big metropolis. Despite recognizing the benefits of the conventional health care, the interviewees opted for maintaining the use of certain medicinal plants, in addition to the replacement and incorporation of novel species, with slower incorporation of species from the native local forest. On the other hand, the maintenance of traditional uses by the population over time demonstrates the high cultural value of the ethnomedical application of these species, suggesting that their potential as pharmacological agents should be evaluated.

## Additional file

Additional file 1: Brazilian books consulted in order to obtain data on their geographical distribution and used to classify the species in native, naturalized or exotic. List of supplementary bibliography consulted. (DOCX 14 kb)

## Abbreviations

BA: Migrants from the state of Bahia
ICF: Informant’s Consensus Factor
PI: Migrants from the state of Piauí
RI: Index of relative importance
