# Supplementary material for: Factors affecting the use of medicinal plants by migrants from rural areas of Brazilian Northeast after moving to a metropolitan region in Southeast of Brazil
Source: J Ethnobiol Ethnomed. 2018 Nov 22;14:72. doi: 10.1186/s13002-018-0270-3 (PMC6249753; doi:10.1186/s13002-018-0270-3)
Supplement: Supplementary file 1 — Brazilian books consulted in order to obtain data on their geographical distribution and used to classify the species in native, naturalized or exotic. List of supplementary bibliography consulted. (DOCX 14 kb) [file 13002_2018_270_MOESM1_ESM.docx]

**Additional file 1:** Brazilian books consulted in order to obtain data on their geographical distribution and used to classify the species in native, naturalized or exotic.

Pio-Correa M. Dicionário de plantas úteis do Brasil e das exóticas cultivadas. Rio de Janeiro: Ministério da Agricultura – IBDF; 1926.

Lorenzi H. Árvores brasileiras: manual de identificação e cultivo de plantas arbóreas nativas do Brasil. V1. Nova Odessa: Plantarum; 1992.

Lorenzi H. Árvores brasileiras: manual de identificação e cultivo de plantas arbóreas nativas do Brasil. V2. Nova Odessa: Instituto Plantarum de Estudos da Flora; 1998.

Lorenzi H, Matos FJA. Plantas medicinais no Brasil: nativas e exóticas. Nova Odessa: Plantarum; 2002.

Lorenzi H, Souza HM, Torres MAV, Bacher LB. Árvores exóticas no Brasil: madeireiras, ornamentais e aromáticas. Nova Odessa: Instituto Plantarum de Estudos da Flora; 2003.
